# White blood cell differential count of maturation stages in bone marrow smear using dual-stage convolutional neural networks

**DOI:** 10.1371/journal.pone.0189259

**Published:** 2017-12-11

**Authors:** Jin Woo Choi, Yunseo Ku, Byeong Wook Yoo, Jung-Ah Kim, Dong Soon Lee, Young Jun Chai, Hyoun-Joong Kong, Hee Chan Kim

**Affiliations:** 1 Interdisciplinary Program in Bioengineering, Graduate School, Seoul National University, Seoul, Korea; 2 Department of Laboratory Medicine, Seoul National University College of Medicine, Cancer Research Institute, Seoul, Korea; 3 Department of Surgery, Seoul National University Boramae Medical Center, Seoul, Korea; 4 Department of Biomedical Engineering, Chungnam National University College of Medicine, Daejeon, Korea; 5 Department of Biomedical Engineering, Seoul National University College of Medicine, Seoul, Korea; 6 Department of Biomedical Engineering, Seoul National University Hospital, Seoul, Korea; 7 Institute of Medical and Biological Engineering, Medical Research Center, Seoul National University, Seoul, Korea; City University London, UNITED KINGDOM

## Abstract

The white blood cell differential count of the bone marrow provides information concerning the distribution of immature and mature cells within maturation stages. The results of such examinations are important for the diagnosis of various diseases and for follow-up care after chemotherapy. However, manual, labor-intensive methods to determine the differential count lead to inter- and intra-variations among the results obtained by hematologists. Therefore, an automated system to conduct the white blood cell differential count is highly desirable, but several difficulties hinder progress. There are variations in the white blood cells of each maturation stage, small inter-class differences within each stage, and variations in images because of the different acquisition and staining processes. Moreover, a large number of classes need to be classified for bone marrow smear analysis, and the high density of touching cells in bone marrow smears renders difficult the segmentation of single cells, which is crucial to traditional image processing and machine learning. Few studies have attempted to discriminate bone marrow cells, and even these have either discriminated only a few classes or yielded insufficient performance. In this study, we propose an automated white blood cell differential counting system from bone marrow smear images using a dual-stage convolutional neural network (CNN). A total of 2,174 patch images were collected for training and testing. The dual-stage CNN classified images into 10 classes of the myeloid and erythroid maturation series, and achieved an accuracy of 97.06%, a precision of 97.13%, a recall of 97.06%, and an F-1 score of 97.1%. The proposed method not only showed high classification performance, but also successfully classified raw images without single cell segmentation and manual feature extraction by implementing CNN. Moreover, it demonstrated rotation and location invariance. These results highlight the promise of the proposed method as an automated white blood cell differential count system.

## Introduction

The differential count of white blood cells (WBCs) is an essential examination in clinical hematology that is conducted on peripheral blood and bone marrow smears. Information obtained from these assessments is used for such purposes as the diagnosis of leukemia, lymphoma, myeloma, myeloproliferative neoplasm, and anemia, and for follow-up care after chemotherapy [1]. This important examination is still manually performed by trained hematologists. They assess the characteristics of cells, such as size, shape, and granularity, using a light microscope. Therefore, the process is not only tedious and labor intensive, but also vulnerable to many sources of error. Intra- and inter-cell variations exist because the morphological characteristics of cells differ within a patient and among patients. Image properties, such as color and contrast, also vary among samples due to the methods used for staining as well as the quality of image acquisition. These make it difficult to obtain an accurate count of WBCs. Since the results are qualitative and highly dependent on the hematologist’s skill and experience, variations within the results obtained by a hematologist, as well as those among measurements by several experts, are unavoidable [2, 3]. In order to solve these problems, a quantified automated analysis system is highly demanded [3–5].

A number of studies have been conducted on automated WBC differentiation in a peripheral blood smear, and commercial computer-aided diagnosis (CAD) systems are available for this purpose [3, 6]. However, an automated WBC differential count of bone marrow smears is problematic and has not been sufficiently researched. Classification of WBCs in bone marrow smears is complex and challenging. In peripheral blood smears, five fully maturated WBCs—basophil, eosinophil, segmented neutrophil, monocyte, and lymphocyte—are usually observed and analyzed. These WBC types have distinct characteristics, so they are relatively easier to discriminate. However, bone marrow smears are typically used to consider the maturation stages of the WBCs. These stages involve more cell types, such as myeloblast, promyelocyte, myelocyte, metamyelocyte, band neutrophil, segmented neutrophil, pronormoblast, basophilic normoblasts, polychromatic normoblast, orthochromatic normoblast, lymphoblast, lymphocyte, monocyte, basophil, eosinophil, and plasma cell. In the diagnosis of hematologic diseases, knowing the ratio of these immature and mature cell types is necessary [6, 7]. Not only do more types of cells need to be discriminated, these stages of maturation are also challenging in the context of defining discrete standards for each cell type, because small inter-class differences exist among continuous stages [8]. Moreover, the cell density of WBCs in the bone marrow smears is higher than that in peripheral blood smears. Due to the high density of bone marrow smears, many WBCs touch one another, which makes it difficult to segment single cells. This is critical in developing an automated WBC differential counter using image processing and traditional machine learning methods, since single-cell segmentation is required for feature extraction and classification [3, 9]. Despite the importance of bone marrow analysis and the high demand for a quantified automated bone marrow smear analyzer, these difficulties hinder progress, and have only been addressed in a few studies.

Attempts have been made in research to classify WBCs in bone marrow smears using image processing and machine learning algorithms. These traditional methods follow the sequence of segmentation, feature extraction, and classification. Many such studies only focused on single-cell segmentation from a bone marrow smear image. Since traditional methods heavily depend on extracted features from segmented single-cell images, the performance of the segmentation algorithm is crucial. Past studies have used several methods for segmentation, such as intensity clustering, watershed transform and adaptive thresholding, support vector machine (SVM), artificial neural network (ANN), simulated visual attention, and many others [3, 10–15]; yet, the segmentation problem has not been perfectly resolved, especially in case of touching cells [6].

In contrast to the above, relatively few studies have been devoted to the classification of the WBCs in bone marrow images. In Theera-Umpon et al. [6], WBCs in bone marrow smear images were classified using the morphological granulomere of the nucleus for six myeloid series. The study used Bayes and ANN classifiers on four extracted features from the nucleus. This approach achieved an accuracy of 63.3% and 65.7% using Bayes and ANN, respectively. It attempts to overcome the problem of touching cells in high-density bone marrow smears by segmenting only the nuclei. This reduced the error rate in cell segmentation, but led to many clinically significant features of the cytoplasm being discarded, hence yielding insufficient classification accuracy. Moreover, the work of Reta et al. [9] showed that features extracted from the nucleus and the cytoplasm are powerful. Several other studies used different methods, such as the multilayer perceptron, the SVM, and feed-forward neural networks for classification [16–21]. Osowski et al. [8] increased the classification accuracy to 83.2% on 11 classes of white blood cells using an SVM and a genetic algorithm. More recently, Staroszczyk et al. [22] showed that using an ensemble of classifiers is more effective to this end. A model combining different feature selection methods and an SVM for an ensemble was proposed, and improved the accuracy to 85.7%. Attempts have also been made to use a CAD system for classifying WBCs of different maturation stages [23–26]. Lee et al. [23] and Briggs et al. [25] used CellaVision DM 96 (CellaVision AB, Sweden) to classify six maturation stages of the myeloid series: blasts, promyelocytes, myelocytes, metamyelocytes, band neutrophils, and segmented neutrophils. In these studies, the CAD system yielded a correlation of 0.86 and 0.74, respectively, with results obtained by expert hematologists. The CAD system was much quicker than the experts, but its performance was unsatisfactory. These studies have been unable to solve the problem of touching cells in high-density bone marrow smears, and have not achieved a desirable accuracy for WBC classification in multiple stages of maturation. These limitations of traditional image processing and machine learning methods are difficult to overcome because of their low learning capacities and their use of handcrafted features.

Deep learning has been spotlighted in machine learning research due to advancements in parallel computation using GPUs, large datasets, and algorithms, the essential ingredients of deep learning. In contrast to traditional image processing and machine learning, deep learning algorithms incorporate feature extraction and classification. Therefore, it can be applied to raw data with minimal pre-processing, such as mean and standard deviation normalization of datasets; deep learning algorithms can also learn more features than handcrafted methods. Deep learning has shown outstanding performance in the classification and recognition of images and signals [27–32]. Its application to medical images has only recently been actively studied. However, the number is increasing rapidly and it is demonstrating astounding performance in various applications, such as mitosis detection from breast cancer pathology images [33, 34], lung cancer detection and classification from CT scans [35, 36], skin cancer classification [37, 38], and diabetic eye disease classification [39]. Kainz *et al*. studied bone marrow cell classification using deep learning, which is the only previous research that applied deep CNN in white blood cell classification [40]. They proposed rotation-invariant WBC classification on a raw image using a recurrent neural network. Approximately 157 images were augmented to 944 images in five classes (four WBC types and one background) for training and testing. The method achieved an accuracy of 96.4% but had some limitations. The background class, which achieved 100% accuracy, should be excluded from WBC classification accuracy for fair comparison, since background images are distinct from WBC images. Moreover, this study tried to discriminate WBCs in the maturation stages, but the number of cell types was not sufficiently large for clinical practice. Further, it required a long processing time and a large amount of computation as the images had to be rotated at every degree. Time and computational effort could have been saved by reducing the frequency of rotation, but this would have come at the cost of classification accuracy.

In this study, we propose an automatic bone marrow WBC differential counter using a convolutional neural network (CNN). The CNN is a deep learning algorithm that has shown strong performance in image recognition and classification [27, 41, 42]. With sparse interactions, parameter sharing, and equivalent representation, the CNN can learn multi-level features from minimally processed raw data and detect complex interactions among the features. Therefore, we aim to exploit the CNN to classify WBCs of the myeloid and erythroid series. The proposed method does not require single-cell segmentation or hand-crafted feature extraction. WBC images were collected and labeled, and trained and tested to classify 10 WBC cell types in different stages of maturation. The proposed method also solves the problem of imbalanced data through oversampling and augmentation, and improves classification performance through the dual-stage network of a global and a local model.

## Methods

### Dataset description

#### Data collection and preparation

The dataset is an important ingredient of the deep learning algorithm, because of which collecting correctly labeled data is essential. However, a large open dataset is unavailable for WBCs, especially for bone marrow smears. Therefore, a dataset was collected for this study. Bone marrow smear samples were prepared at the Department of Laboratory Medicine, Seoul National University Hospital. The bone marrow aspiration involved staining using the Wright-Giemsa protocol. The images of the prepared slides were acquired with a light microscope at x1000 magnification. For each slide, five to ten non-overlapping acquisition locations were randomly selected, and a total of 200 images with a pixel resolution of 1080 x 1330 were obtained from 30 slides of 10 human subjects. The preparation of images were conducted anonymously using previously-collected data. The study protocol received exempt status from the institutional review board at the Seoul National University Hospital.

**Fig 1:**
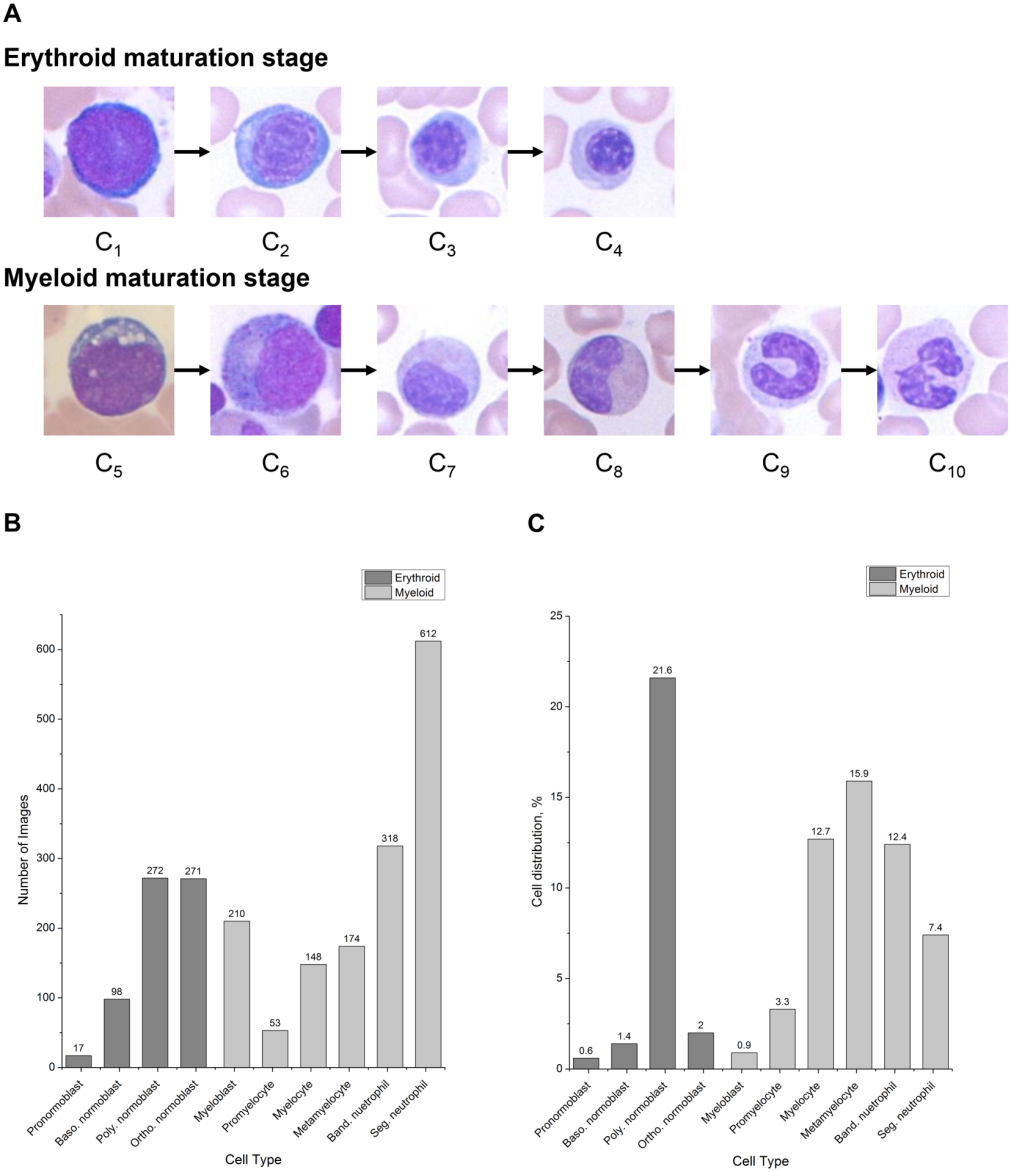
Description of collected data. (A) Examples of white blood cells in erythroid series (C_1-4_) and myeloid series (C_5-10_). (B) Distribution of collected data. (C) Cellular component distribution in bone marrow.

Entire images were manually cropped into 96 x 96-pixel single-cell patch images. A total of 2,174 cropped images were collected; these single-cell images were labeled and confirmed as having been placed in the correct classes by two expert hematologists. The dataset was composed of 10 WBC classes in the stages of maturation, including four consecutive stages of the erythroid series—pronormoblast (C_1_), basophilic normoblast (C_2_), polychromatic normoblast (C_3_), and orthochromatic normoblast (C_4_)—and six consecutive stages of the myeloid series—myeloblast (C_5_), promyelocyte (C_6_), myelocyte (C_7_), metamyelocyte (C_8_), band neutrophil (C_9_), and segmented neutrophil (C_10_). The examples of WBCs in the stages of maturation are shown in Fig 1A. The cells in the same series had high intra-class differences due to the continuous maturation process.

A number of images for each class and distribution are shown in Fig 1B. The collected dataset had an imbalanced distribution among classes. This problem was unavoidable while collecting the dataset, since the natural distribution of white blood cells is imbalanced, as shown in Fig 1C. However, this imbalanced dataset can be a problem in training the network, as only a few features are learned from classes with relatively small number of data [43].

#### Data oversampling and augmentation

**Fig 2:**
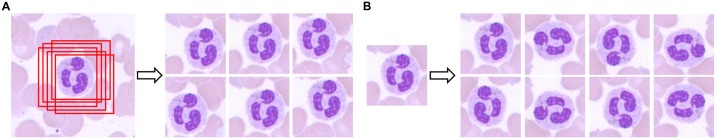
Examples of data preparation. (A) Oversampling and (B) Augmentation.

In an attempt to resolve the problem of imbalanced data due to the heterogeneous distribution of white blood cells, oversampling was conducted for classes with relatively small numbers of data items. During data preparation, we manually cropped multiple images of the same cells at slightly different centers for classes with few data. This increased the number of data and provided more diverse data. Examples of oversampled images are shown in Fig 2A.

In order to solve the problem of the rotation variation in white blood cell classification, the dataset was augmented by a factor of eight. Image patches were transformed through combinations of four angles of rotation (0°, 90°, 180°, 270°) and two flips (horizontal and vertical). We ensured that no duplication existed between the original and augmented images. Augmentation solves the problem of rotation variation and increases the number of data to help network training. Examples of augmented images are shown in Fig 2B.

### Convolutional neural network

#### Architecture and two-stage CNN

**Fig 3:**
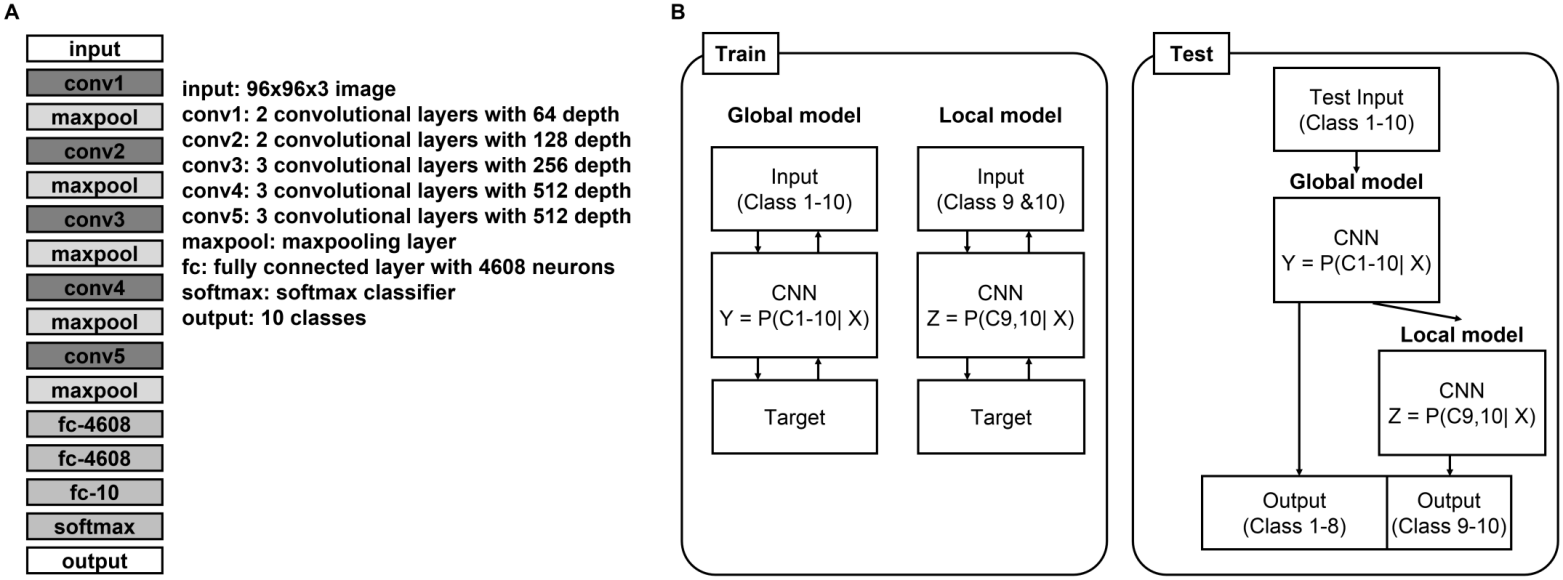
Description of networks. (A) Illustration of the convolutional neural network. (B) Description of the proposed dual-stage convolutional neural network.

The architecture of the CNN was inspired by VGGnet developed by Simonyan *et al*. [44]. The network used in this study was composed of 16 layers, 13 convolutional layers with max-pooling, and three fully connected layers followed by a softmax classifier layer. The dimensions of the input were set to 96 x 96 x 3 and 3 x 3 filters were used for the convolutional layers. The max-pooling layers were operated in 2 x 2 regions with a stride of 2. In order to prevent overfitting of the trained network, a dropout [45] was placed between all pairs of convolutional layers. The dropout randomly deactivated some weights in the convolutional layer; the dropout ratio was set in the range 0.3 to 0.5. Batch normalization was implemented after the convolutional layers with a batch size of 40 to prevent overfitting. Moreover, a rectified linear-unit (ReLU) activation function [27] was used following each instance of batch normalization for effective learning and fast convergence. The network architecture is shown in Fig 3A.

We propose a dual-stage CNN with a global and a local model. The last fully connected layer of the local model was changed from ten to two. A global model of 10 classes and a local model of two classes were trained and combined, as shown in Fig 3B, for testing. This customized architecture for the WBC differential count was designed to fine-tune cases where the global model incorrectly classifies two consecutive maturation stages. In this study, we trained the local model for the band neutrophils and segmented neutrophils classes, which are difficult to classify due to their complex nuclear shapes and granularities. It is widely acknowledged that band cells cannot be reliably distinguished from segmented neutrophils on blood films by human observers [46, 47].

#### Training of CNN

The weights of each layer were initialized using the MSRA method [48]. It randomly assigns weights from a zero-mean Gaussian distribution with a standard deviation of $\sqrt{\text{2}/{\text{N}}_{\text{in}}}$, where Nin is the number of inputs to a neuron. Following initialization, the network was trained and optimized through stochastic gradient descent to minimize the cross-entropy loss function:

$$Loss:L=\frac{1}{N}\sum_{i}D(S_{i},T_{i})$$ (1)

where *N* is a total number of images, and *i* is the i-th single image of a trained dataset. *S* is an output vector of the softmax classifier that assigns probabilities of classes between 0 and 1, and *T* is a target label vector of the image, where 1 is assigned for a correct assignment to a class and 0 for incorrect assignments. The cross-entropy of each image was computed as follows:

$$Crossentropy:D(S,T)=-\sum_{c}T_{c}\;\text{log}S_{c}$$ (2)

$$Softmax:S(y_{c})=\frac{e^{y_{c}}}{\sum_{j}e^{y_{j}}}$$ (3)

where *c* denotes a class among many, *y* denotes a vector of the class output scores of a network, and *j* also denotes classes.

The prepared image dataset was divided into training-validation and testing datasets with a ratio of 5:1. The training-validation dataset was further divided into a ratio of 4:1 ratio for five-fold cross-validation, and was used to train and optimize the hyper-parameters: learning ratio, momentum, learning rate decay, and weight decay. The images of each dataset were transformed from the RGB color channel to YUV. The training dataset was normalized using the mean and standard deviation of each channel. The mean and standard deviation of the training dataset were recorded to normalize the validation and test datasets. The networks were trained from scratch without any pre-training for 150 epochs.

To assess the effects of the number of data, the augmentation, and oversampling, the network was trained using six datasets; the original dataset, the augmented dataset, the dataset oversampled to 300 images per class, oversampled to 600 images per class, the dataset augmented and oversampled to 300 images per class, and that augmented and oversampled to 600 images per class. These datasets contained 2,174, 17,392, 3,000, 6,000, 24,000, and 48000 images, respectively. For these cases, only the training dataset was augmented and/or oversampled, and the test dataset was used only for performance evaluation. To analyze the location invariance and the rotation invariance of the trained networks, randomly selected images from the band neutrophil and segmented neutrophil test datasets were oversampled and augmented. Moreover, the dual-stage CNN was constructed using the datasets augmented and oversampled to 600 images per class, which yielded the best performance, as the global model and the local model of classes 9 and 10, which corresponds to band neutrophil and segmented neutrophil.

#### Implementation

The proposed method was implemented using the Torch7 framework and the CUDA toolkit with the cuDNN library on Linux OS. All experiments were performed with a CPU i7-6700 (3.40 GHz), RAM 16 GB, and GPU NVIDIA GTX 980 (4 GB).

#### Evaluation metrics

The trained network was tested on the test dataset and classification performance was assessed quantitatively through the following metrics: mean accuracy, precision, recall, and F1 score,

$$\begin{array}{l} Accuracy=\frac{T_{P}+T_{N}}{T_{P}+F_{P}+T_{N}+F_{N}}\\ Precision=\frac{T_{P}}{T_{P}+F_{P}}\\ Recall=\frac{T_{P}}{T_{P}+F_{N}}\\ F1-score=2\cdot \frac{Precision\cdot Recall}{Precision+Recall} \end{array}$$ (4)

where *T*_P_ is the number of true positive classifications, *T*_N_ the number of true negatives, *F*_P_ is the number false positive classifications, and *F*_N_ the number of false negatives. A confusion matrix of the classes was also created to analyze class-wise performance.

## Results and discussion

Experiments were conducted on the six datasets, and the classification performance of the proposed method was compared for the six datasets according to the above evaluation metrics. The classification performance of the network trained on original data (OG network), augmented data (AG network), oversampled data with 300 images per class (OS 300 network), oversampled data with 600 images per class (OS 600 network), augmented and oversampled data with 300 images per class (AG+OS 300 network), and augmented and oversampled data with 600 images per class (AG+OS 600 network) is summarized in Table 1.

**Table 1:** Classification performance of the network trained on different datasets.

Dataset	Accuracy	Precision	Recall	F1 score
**Original**	57.80	83.36	48.69	61.47
**Augmentation**	71.90	65.17	65.00	65.08
**Oversampling 300**	65.62	68.59	65.61	67.07
**Oversampling 600**	90.57	91.04	90.57	90.80
**Augmentation + Oversampling 300**	85.05	85.02	85.05	85.04
**Augmentation + Oversampling 600**	95.68	95.49	95.68	95.58

The accuracy of the OG network yielded the worst accuracy at 57.8%, but the AG network and the OS 300 network recorded slightly higher accuracy values of 71.9% and 65.62%, respectively. The OS 600 network and the AG+OS 300 network significantly improved classification performance with accuracies of 90.57% and 85.05%, respectively. The AG+OS 600 network demonstrated the best performance with a 95.68% accuracy, which was as good as the state-of-art method of Kainz *et al*. It is widely accepted that deep learning algorithms train better networks with larger amounts of data, and the result showed a similar trend in general, except for the OS 600 network. This network, which was trained on 6,000 images, showed better performance to the AG+OS 300 and the AG networks, which were trained on 24,000 and 17,392 images, respectively. This indicates that the diversity of data, in addition to the total number of data, is also important in training the CNN for WBC differential count. Most cell types have round-shaped cells and nuclei; thus, the rotational augmentation of images was less effective in creating diverse data than oversampling. Hence, while augmentation is widely used to increase the number of data in deep learning in general, this may not be effective or practical for WBC images. Rescaling augmentation cannot be applied to WBC classification because the size of cell is an important characteristic. Moreover, in other medical images, even rotational augmentation is not acceptable, since the orientations of images represent important information. However, augmentation was able to improve performance with the same number of oversampled data. The precision, the recall, and the F1 score also showed similar trends as accuracy, such that the AG+OS 600 recorded the best performance. The problems of an imbalanced dataset and low performance were solved through data oversampling and augmentation.

**Fig 4:**
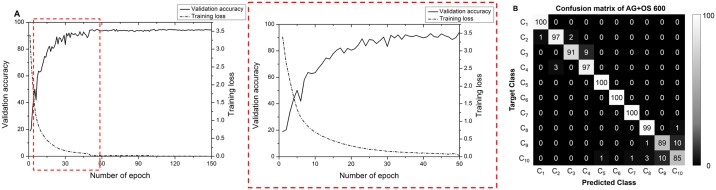
Details of training networks. (A) Graph of validation accuracy and training loss during training of network. The dotted red box shows the magnified view of the first 50 epochs. (B) Confusion matrix of AG+OS 600.

Further analysis was conducted on the AG+OS 600 network. To confirm that it had been successfully trained, the accuracy of the validation dataset and training loss were plotted over 150 epochs (Fig 4A). Accuracy converged after approximately 100 epochs, when the training loss also converged to 0.004. The final trained network achieved 99.7% accuracy on the training dataset and 96% on the validation dataset. A confusion matrix of the classification results on the test dataset was generated to evaluate class-wise performance (Fig 4B). The first four classes represent WBCs of the erythroid maturation series and the last six the WBCs of the myeloid maturation series. As shown in the confusion matrix, misclassifications occurred within the same maturation series, mostly within the consecutive maturation stage. A majority of misclassifications occurred in the band neutrophil and the segmented neutrophil, which achieved 89% and 85% in terms of recall, respectively.

**Fig 5:**
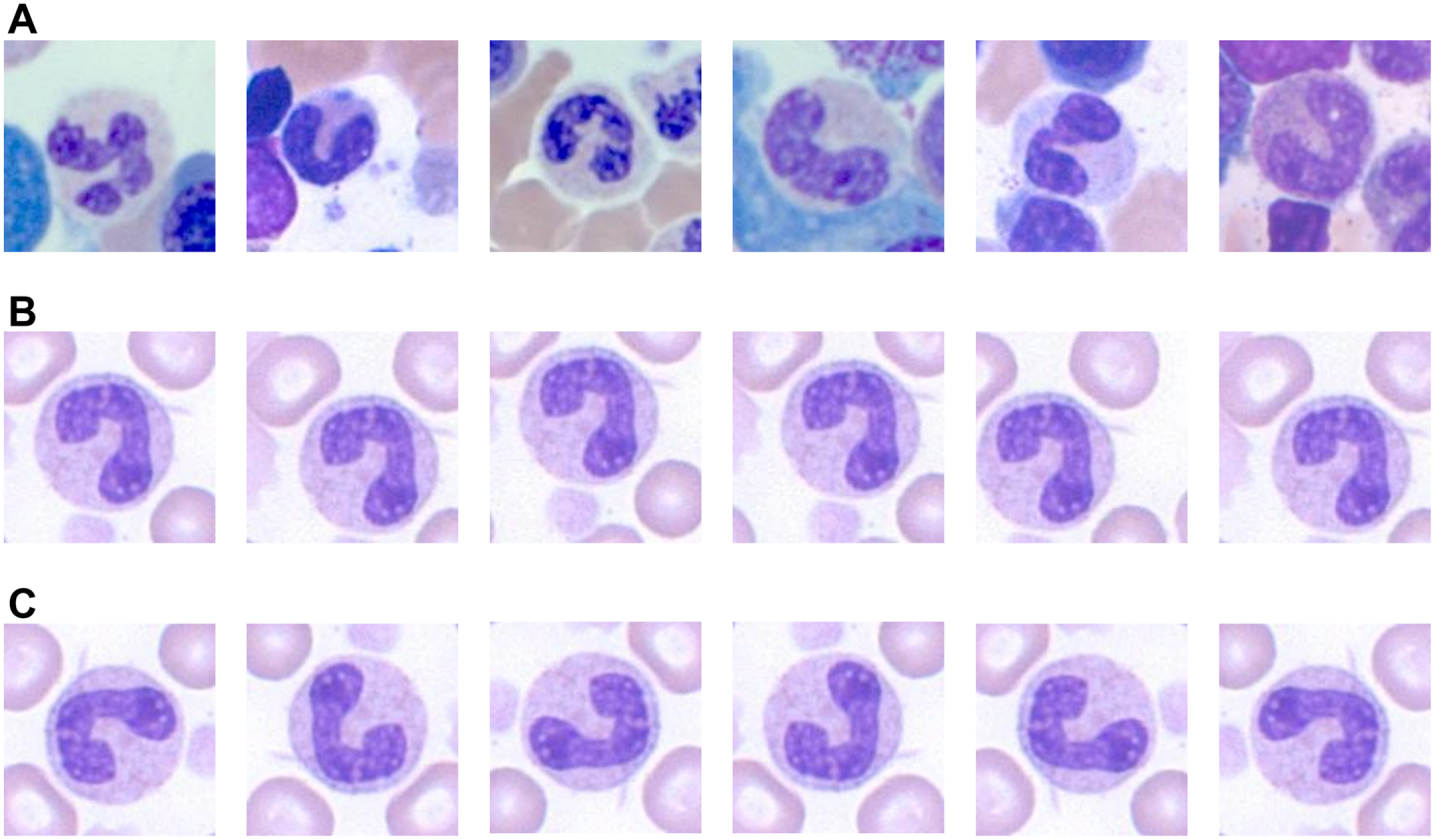
Examples of correctly classified cells by the AG+OS 600 network. (A) WBCs with backgrounds showing background invariance of the network. (B) Oversampled WBCs showing location invariance of the network. (C) Augmented WBCs showing rotation invariance of the network.

The classification of band neutrophil and segmented neutrophil was explored in greater detail on the AG+OS 600 network. Fig 5 shows the correctly classified images of the band neutrophil and the segmented neutrophil. The network was able to classify images with backgrounds, as shown in Fig 5A. These images not only contained red blood cells and parts of other WBCs in the background, but also featured cells touching the target cells. This result indicates that the network was background invariant, and can be applied to raw images without WBC segmentation, which is a problem in traditional image processing and machine learning methods. Moreover, a randomly selected image from the band neutrophil and the segmented neutrophil test datasets was oversampled and tested to assess the network’s location invariance (Fig 5B). The AG+OS 600 network was able to correctly classify the oversampled images. To ensure that this was due to data oversampling, we tested the same oversampled images on other networks. The AG network was not able to classify most of these oversampled images, whereas the OS network correctly classified a few. This location-invariant network can help develop a combined system of detection and classification. If the detection algorithm slightly misses the center of the cell, the trained model can still deliver good performance. The augmented images were also assessed to show that the network was rotation invariant. A randomly selected image from the band neutrophil and the segmented neutrophil test datasets was augmented and tested (Fig 5C). The AG+OS 600 network was able to correctly classify the augmented images. To ensure that this was the consequence of data augmentation, we tested the same augmented images on other networks. The OS network was not able to classify most augmented images, whereas the AG network correctly classified a few. We can thus conclude that data augmentation is effective in training a rotation-invariant network.

**Fig 6:**
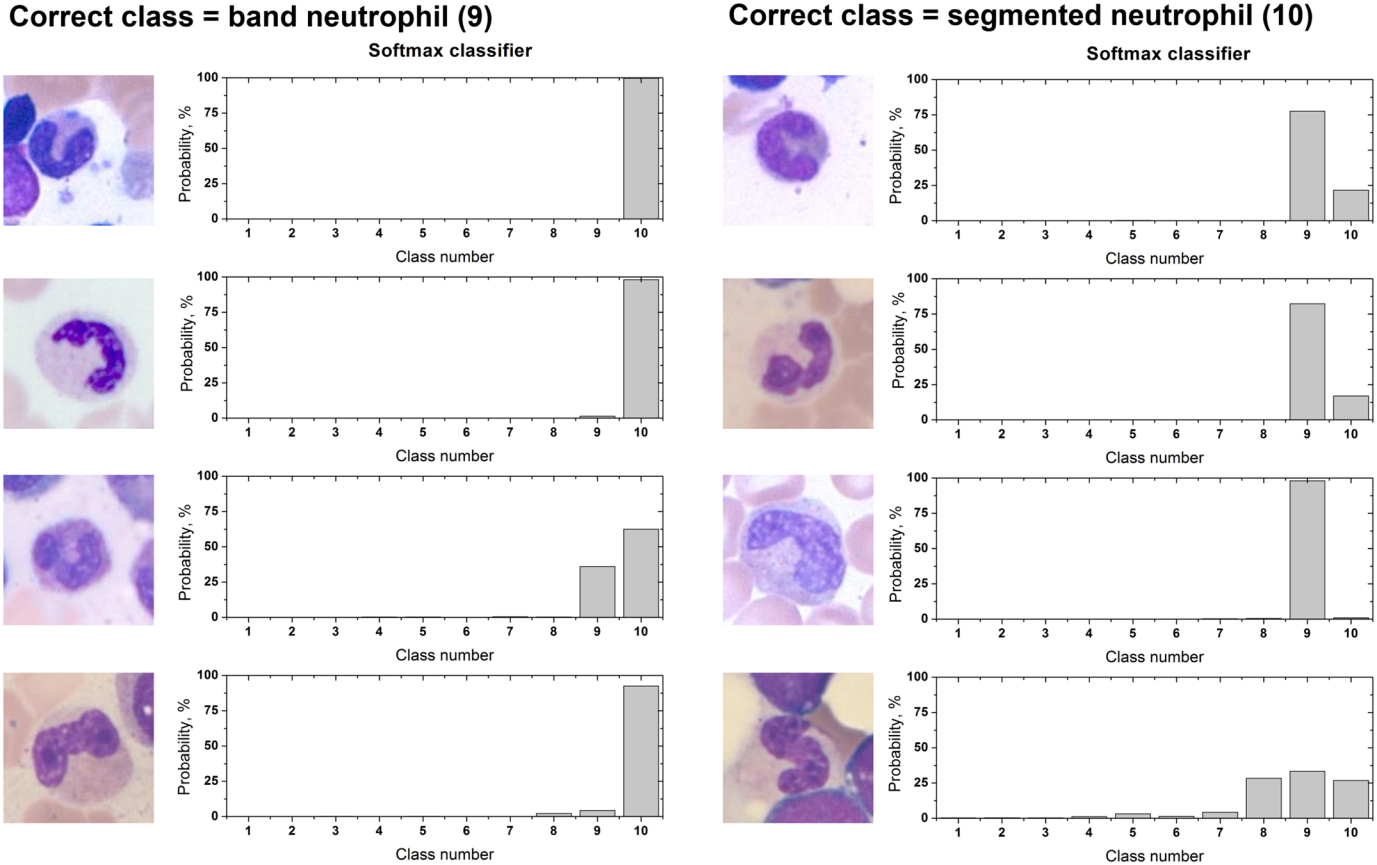
Examples of incorrectly classified cells by the AG+OS 600 network. (A)-(D) Cells whose ground truth is band neutrophil. (E)-(H) Cells whose ground truth is segmented Neutrophil.

Incorrectly classified images of the band neutrophil and the segmented neutrophils were also analyzed. Some cases are shown in Fig 6 with probability distribution results from the softmax classifier. The deep learning algorithm predicted the classes according to the probability of the softmax classifier. The correct class in the left column of Fig 6 is band neutrophil; yet, the network predicted these images as belonging to the segmented neutrophil. The correct class on the right column of Fig 6 is the segmented neutrophil; yet, the network predicted the images as belonging to the band neutrophil.

Although, the data collection was carefully conducted over almost a year by two experts who were trained over 7 years, the process of data collection is tedious and error-prone, so these misclassified cells were confirmed by a third expert. There were controversies on few cases, for example, the third expert had different opinion on the last two rows of both band neutrophil and segmented neutrophil in Fig 6C, 6D, 6G and 6H. The person commented that these cells are on a board between band neutrophil and segmented neutrophil. Specifically, the cell of Fig 6C should be labeled as segmented neutrophil and the cell of Fig 6G should be labeled as band neutrophil. The expert was uncertain about Fig 6D and 6H. However, the maturation of white blood cell is a continuous process, so it is difficult to provide discrete discriminative standards and there may exist different opinions. This demonstrates the issue of differential count, which highly depends on the experts’ opinions and experiences.

**Fig 7:**
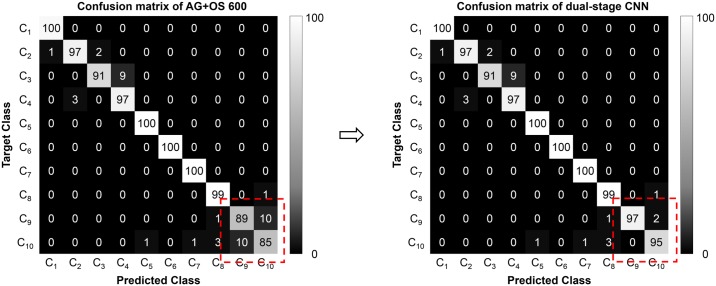
Comparison of confusion matrices of AG+OS 600 and dual-stage CNN.

The confusion matrix of the dual-stage CNN classification for the 10 classes considered on the test dataset is shown in Fig 7. The dual-stage CNN was able to correct the misclassifications in the band neutrophil and the segmented neutrophil of the AG+OS 600 network as shown in the red dotted boxes of Fig 7. The dual-stage CNN achieved a 97.06% accuracy, a 97.13% precision, a 97.06% recall, and a 97.1% F1 score. It thus outperformed any previously reported research for cases involving a large number of WBC types, which had hitherto yielded accuracy values below 90%. It even surpassed the 96.4% accuracy of the state-of-the-art method proposed by Kainz *et al*, which only classified four WBC types. Moreover, the dual-stage CNN classified images with backgrounds, augmented images, and oversampled images in Fig 5 as well, which indicates that it is rotation and location invariant, and can be applied to raw images.

The proposed work focused on WBC classification and yielded impressive performance. However, there is some room for improvement from the perspective of the entire system. The automated WBC differential counting system should include a detection process for high-content screening. Such screening has been used computer-aided diagnosis with a high-resolution microscopic scanner [4, 49]. This can help reduce time, cost, and labor, and can enhance the accuracy of diagnosis by assessing a larger number of cells in a bone marrow smear slide. Moreover, the proposed method can discriminate 10 WBCs of the maturation stages, but it should include a complete list of WBC types, such as plasma cells, lymphocytes, and specific disease models, such as stages of leukemia, should be included as well. Being able to classify the complete list of cell types and diseases is important, but a dataset that can be used for this purpose does not yet exist. Therefore, a dataset of complete WBC types should first be created with reliable ground truths. Training of CNN highly depends not only on the size of data, but also on the reliability of ground truth labels. However, the process of collecting data is very tedious task and is error-prone. Moreover, it is difficult to separate continuous maturation stages with discrete discriminative standards as mentioned previously. So, the dataset should be carefully prepared with these considerations and a protocol that can collect reliable data without any controversies should be developed. Lastly, it is necessary to design an architecture that is specifically for WBC differential count. VGGnet, the network that was used in this study, was originally designed for a general purpose classification of natural images. Therefore, designing an architecture for this specific purpose would improve the performance, and the new architecture should be validated by comparing with other existing networks.
